# 1-Bromo-2-chloro-4,5-dimethoxy­benzene

**DOI:** 10.1107/S1600536810008445

**Published:** 2010-03-13

**Authors:** Yang Song, Sean Parkin, Hans-Joachim Lehmler

**Affiliations:** a College of Pharmaceutical Sciences, Key Laboratory of Eco-environments in Three Gorges Reservoir Region, Ministry of Education, Southwest University, Chong Qing 400716, People’s Republic of China; b University of Kentucky, Department of Chemistry, Lexington, KY 40506-0055, USA; c The University of Iowa, Department of Occupational and Environmental Health, 100 Oakdale Campus, 124 IREH, Iowa City, IA 52242-5000, USA

## Abstract

The two meth­oxy groups of the title compound, C_8_H_8_BrClO_2_, are approximately coplanar with the benzene ring, the dihedral angles in all four mol­ecules in the asymmetric unit ranging from of 0.9 (3) to 12.3 (3)°. All four independent mol­ecules are disordered by different amounts about non-crystallographic twofold axes which nearly superimpose the Cl and Br sites.

## Related literature

For similar structures of halogenated meth­oxy benzenes, see: Iimura *et al.* (1984 ▶); Rissanen *et al.* (1987 ▶, 1988*a* ▶,*b* ▶); Song *et al.* (2008 ▶, 2010*a* ▶,*b* ▶); Telu *et al.* (2008 ▶); Weller & Gerstner (1995 ▶); Wieczorek (1980 ▶). For general background to halogenated meth­oxy benzenes, see: Ballschmiter, (2003 ▶); Brownlee *et al.* (1993 ▶); Curtis *et al.* (1972 ▶); Pereira *et al.* (2000 ▶); Vlachos *et al.* (2007 ▶).
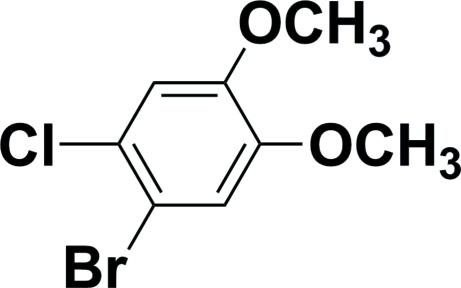


## Experimental

### 

#### Crystal data

C_8_H_8_BrClO_2_
*M* *_r_* = 251.50
Triclinic, 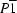

*a* = 9.9264 (2) Å
*b* = 9.9410 (2) Å
*c* = 19.7219 (5) Å
α = 75.9259 (8)°
β = 75.9323 (8)°
γ = 79.9479 (10)°
*V* = 1817.26 (7) Å^3^
*Z* = 8
Mo *K*α radiation
μ = 4.77 mm^−1^
*T* = 90 K
0.22 × 0.22 × 0.20 mm

#### Data collection

Nonius KappaCCD diffractometer
Absorption correction: multi-scan (*SCALEPACK*; Otwinowski & Minor, 1997 ▶) *T*_min_ = 0.360, *T*_max_ = 0.385
14754 measured reflections
8202 independent reflections
6065 reflections with *I* > 2σ(*I*)
*R*_int_ = 0.035

#### Refinement

*R*[*F* ^2^ > 2σ(*F* ^2^)] = 0.036
*wR*(*F* ^2^) = 0.070
*S* = 1.04
8202 reflections
471 parameters
16 restraints
H-atom parameters constrained
Δρ_max_ = 0.43 e Å^−3^
Δρ_min_ = −0.42 e Å^−3^

### 

Data collection: *COLLECT* (Nonius, 1998 ▶); cell refinement: *SCALEPACK* (Otwinowski & Minor, 1997 ▶); data reduction: *DENZO-SMN* (Otwinowski & Minor, 1997 ▶); program(s) used to solve structure: *SHELXS97* (Sheldrick, 2008 ▶); program(s) used to refine structure: *SHELXL97* (Sheldrick, 2008 ▶); molecular graphics: *XP* in *SHELXTL* (Sheldrick, 2008 ▶); software used to prepare material for publication: *SHELXL97* and local procedures.

## Supplementary Material

Crystal structure: contains datablocks I, global. DOI: 10.1107/S1600536810008445/ci5050sup1.cif

Structure factors: contains datablocks I. DOI: 10.1107/S1600536810008445/ci5050Isup2.hkl

Additional supplementary materials:  crystallographic information; 3D view; checkCIF report

## Figures and Tables

**Fig. 1.**
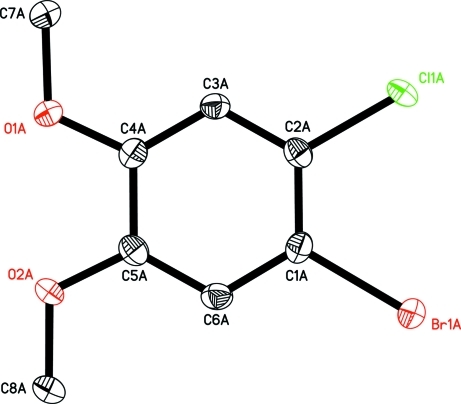
One of the four independent molecules of the title compound, showing the atom-labelling scheme. Displacement ellipsoids are drawn at the 50% probability level. Only the major disorder component is shown.

## supplementary crystallographic information

### Comment

Halogenated methoxy benzenes are an important group of volatile organic pollutants (Ballschmiter, 2003) that cause off-flavors in water, fish, chicken and wine (Brownlee *et al.*, 1993; Curtis *et al.*, 1972; Pereira *et al.*, 2000; Vlachos *et al.*, 2007). Although the conformation of the methoxy groups relative to the aromatic ring system plays an important role in the biological and olfactory properties of this class of compounds, only a few crystal structures of brominated and/or chlorinated methoxy benzenes have been published. Here we report the crystal structure of the title compound, a halogenated dimethoxy benzene, to aid in quantitative structure activity relationship studies.

The asymmetric unit of the title compound contains four independent molecules (A, B, C and D). All methoxy groups are approximately co-planar with the attached benzene ring, with dihedral angles between benzene ring (C1-C6) and methoxy plane (C4/O1/C7 or C5/O2/C8) ranging from 0.9 (3)° to 5.0 (3)°. One exception is the dihedral angle between C1C–C6C and C5C/O2C/C8C planes of 12.3 (3)°. This comparatively large dihedral angle is most likely a result of crystal packing effects. Analogously, the methoxy groups of structurally related compounds with no or one substituent *ortho* to the methoxy group typically lie within the plane of the benzene ring (Rissanen *et al.*, 1988*a*; Song *et al.*, 2010*a*). In contrast, much larger dihedral angles are observed for halogenated methoxy benzenes with two (chlorine) substituents *ortho* to the methoxy group (Rissanen *et al.*, 1987, 1988*b*; Song *et al.* 2010*b*; Telu *et al.*, 2008; Weller & Gerstner, 1995; Wieczorek, 1980). For example, in 1-bromo-2,3,6-trichloro-4,5-dimethoxybenzene, a structurally related dimethoxy benzene, the dihedral angles involving the two methoxy groups are much larger [68.5 (3)° and 84.7 (3)°; Song *et al.*, 2010*b*].

### Experimental

The title compound was synthesized by chlorination of 1-bromo-3,4-dimethoxy-benzene with HCl/H_2_O_2_ as chlorination reagent as described previously (Song *et al.*, 2008). Crystals suitable for X-ray diffraction were grown by slow evaporation of a saturated solution of the title compound in CHCl_3_.

### Refinement

H atoms were found in difference Fourier maps and subsequently placed in idealized positions with constrained C–H distances of 0.98 Å (RCH_3_), 0.95 Å (C_Ar_H), and with U_iso_(H) values set to either 1.2U_eq_ or 1.5U_eq_ (RCH_3_) of the attached C atom. Each of the four independent molecules was found to be disordered by a non-crystallographic twofold rotation about an axis running approximately through the bisectors of bonds C1—C2 and C4—C5. This disorder nearly superimposes Cl and Br at the halogen sites. The occupancy ratios for the major and minor components of molecules A, B, C and D are 0.7451 (15):0.2549 (15), 0.5438 (15):0.4562 (15), 0.5027 (15):0.4973 (15) and 0.6246 (15):0.3754 (15), respectively. As a result of the disorder, a number of constraints and restraints were required to ensure that the refinement was stable. The displacement parameters of Cl and Br atoms that are roughly superimposed by the disorder were constrained to be the same. The C—Cl and C—Br distances were restrained using a free variable.

### Crystal data

**Table:**

C_8_H_8_BrClO_2_	*Z* = 8
*M**_r_* = 251.50	*F*(000) = 992
Triclinic, *P*1	*D*_x_ = 1.839 Mg m^−^^3^
Hall symbol: -P 1	Mo *K*α radiation, λ = 0.71073 Å
*a* = 9.9264 (2) Å	Cell parameters from 7193 reflections
*b* = 9.9410 (2) Å	θ = 1.0–27.5°
*c* = 19.7219 (5) Å	µ = 4.77 mm^−^^1^
α = 75.9259 (8)°	*T* = 90 K
β = 75.9323 (8)°	Block, colourless
γ = 79.9479 (10)°	0.22 × 0.22 × 0.20 mm
*V* = 1817.26 (7) Å^3^	

### Data collection

**Table:**

Nonius KappaCCD diffractometer	8202 independent reflections
Radiation source: fine-focus sealed tube	6065 reflections with *I* > 2σ(*I*)
graphite	*R*_int_ = 0.035
Detector resolution: 18 pixels mm^-1^	θ_max_ = 27.5°, θ_min_ = 2.1°
ω scans at fixed χ = 55°	*h* = −12→12
Absorption correction: multi-scan (*SCALEPACK*; Otwinowski & Minor, 1997)	*k* = −12→12
*T*_min_ = 0.360, *T*_max_ = 0.385	*l* = −25→25
14754 measured reflections	

### Refinement

**Table:**

Refinement on *F*^2^	Primary atom site location: structure-invariant direct methods
Least-squares matrix: full	Secondary atom site location: difference Fourier map
*R*[*F*^2^ > 2σ(*F*^2^)] = 0.036	Hydrogen site location: inferred from neighbouring sites
*wR*(*F*^2^) = 0.070	H-atom parameters constrained
*S* = 1.04	*w* = 1/[σ^2^(*F*_o_^2^) + (0.0154*P*)^2^ + 1.1857*P*] where *P* = (*F*_o_^2^ + 2*F*_c_^2^)/3
8202 reflections	(Δ/σ)_max_ = 0.004
471 parameters	Δρ_max_ = 0.43 e Å^−^^3^
16 restraints	Δρ_min_ = −0.41 e Å^−^^3^

### Special details

**Table:**

Experimental. The triclinic cell appears to transform to a C-centered monoclinic cell but the data fail to merge in a satisfactory way in that setting. The structure solved and refined well with the triclinic setting in spite of the extensive disorder. The refined model does not transform to any monoclinic C model either manually or by use of missed symmetry algorithms such as ADDSYM as implemented in Platon (Spek).
Geometry. All esds (except the esd in the dihedral angle between two l.s. planes) are estimated using the full covariance matrix. The cell esds are taken into account individually in the estimation of esds in distances, angles and torsion angles; correlations between esds in cell parameters are only used when they are defined by crystal symmetry. An approximate (isotropic) treatment of cell esds is used for estimating esds involving l.s. planes.
Refinement. Refinement of *F*^2^ against ALL reflections. The weighted *R*-factor wR and goodness of fit *S* are based on *F*^2^, conventional *R*-factors *R* are based on *F*, with *F* set to zero for negative *F*^2^. The threshold expression of *F*^2^ > σ(*F*^2^) is used only for calculating *R*-factors(gt) etc. and is not relevant to the choice of reflections for refinement. *R*-factors based on *F*^2^ are statistically about twice as large as those based on *F*, and *R*- factors based on ALL data will be even larger.

### Fractional atomic coordinates and isotropic or equivalent isotropic displacement parameters (Å^2^)

**Table:**

	*x*	*y*	*z*	*U*_iso_*/*U*_eq_	Occ. (<1)
Br1A	−0.0045 (2)	0.9524 (3)	0.89744 (16)	0.0246 (2)	0.7451 (15)
Cl1A	0.0944 (17)	0.6347 (17)	0.9666 (8)	0.0225 (9)	0.7451 (15)
Br1E	0.096 (2)	0.618 (2)	0.9722 (9)	0.0225 (9)	0.2549 (15)
Cl1E	0.0103 (17)	0.954 (2)	0.8902 (13)	0.0246 (2)	0.2549 (15)
O1A	0.54879 (19)	0.65270 (19)	0.77345 (10)	0.0217 (5)	
O2A	0.47578 (19)	0.90927 (19)	0.71339 (10)	0.0228 (5)	
C1A	0.1671 (3)	0.8534 (3)	0.85998 (15)	0.0192 (6)	
C2A	0.2065 (3)	0.7175 (3)	0.89182 (14)	0.0177 (6)	
C3A	0.3335 (3)	0.6477 (3)	0.86393 (15)	0.0177 (6)	
H3A	0.3599	0.5538	0.8860	0.021*	
C4A	0.4218 (3)	0.7129 (3)	0.80463 (15)	0.0177 (6)	
C5A	0.3819 (3)	0.8520 (3)	0.77156 (15)	0.0189 (6)	
C6A	0.2548 (3)	0.9211 (3)	0.79966 (15)	0.0194 (6)	
H6A	0.2273	1.0148	0.7777	0.023*	
C7A	0.5938 (3)	0.5126 (3)	0.80712 (16)	0.0214 (7)	
H7A1	0.6038	0.5111	0.8555	0.032*	
H7A2	0.6841	0.4794	0.7792	0.032*	
H7A3	0.5243	0.4517	0.8096	0.032*	
C8A	0.4342 (3)	1.0479 (3)	0.67626 (16)	0.0249 (7)	
H8A1	0.3468	1.0494	0.6611	0.037*	
H8A2	0.5076	1.0756	0.6341	0.037*	
H8A3	0.4200	1.1132	0.7081	0.037*	
Br1B	0.4007 (8)	0.3438 (5)	1.0251 (4)	0.0247 (4)	0.5438 (15)
Cl1B	0.698 (2)	0.2075 (19)	0.9410 (8)	0.0245 (6)	0.5438 (15)
Br1F	0.7056 (10)	0.2212 (9)	0.9393 (4)	0.0245 (6)	0.4562 (15)
Cl1F	0.400 (2)	0.3317 (15)	1.0310 (12)	0.0247 (4)	0.4562 (15)
O1B	0.41582 (19)	−0.1413 (2)	1.20683 (10)	0.0233 (5)	
O2B	0.65841 (19)	−0.23454 (19)	1.14057 (10)	0.0219 (5)	
C1B	0.6106 (3)	0.1110 (3)	1.02176 (14)	0.0182 (6)	
C2B	0.4826 (3)	0.1619 (3)	1.05831 (15)	0.0186 (6)	
C3B	0.4143 (3)	0.0807 (3)	1.12061 (15)	0.0201 (6)	
H3B	0.3263	0.1169	1.1455	0.024*	
C4B	0.4733 (3)	−0.0519 (3)	1.14657 (15)	0.0168 (6)	
C5B	0.6059 (3)	−0.1041 (3)	1.10984 (15)	0.0170 (6)	
C6B	0.6721 (3)	−0.0233 (3)	1.04787 (15)	0.0175 (6)	
H6B	0.7602	−0.0588	1.0227	0.021*	
C7B	0.2835 (3)	−0.0911 (3)	1.24616 (16)	0.0291 (7)	
H7B1	0.2936	−0.0102	1.2639	0.044*	
H7B2	0.2503	−0.1652	1.2867	0.044*	
H7B3	0.2159	−0.0635	1.2149	0.044*	
C8B	0.7944 (3)	−0.2893 (3)	1.10553 (16)	0.0247 (7)	
H8B1	0.7907	−0.2980	1.0576	0.037*	
H8B2	0.8227	−0.3815	1.1334	0.037*	
H8B3	0.8624	−0.2259	1.1017	0.037*	
Br1C	1.2543 (5)	0.6563 (7)	0.4287 (3)	0.0254 (4)	0.5027 (15)
Cl1C	1.3717 (15)	0.357 (2)	0.5194 (12)	0.0222 (4)	0.5027 (15)
Br1G	1.3817 (6)	0.3453 (8)	0.5159 (5)	0.0222 (4)	0.4973 (15)
Cl1G	1.2350 (13)	0.6548 (19)	0.4322 (7)	0.0254 (4)	0.4973 (15)
O1C	0.83249 (19)	0.63322 (19)	0.65073 (10)	0.0217 (5)	
O2C	0.9333 (2)	0.3954 (2)	0.71708 (10)	0.0233 (5)	
C1C	1.2099 (3)	0.4433 (3)	0.55384 (15)	0.0174 (6)	
C2C	1.1566 (3)	0.5695 (3)	0.51819 (14)	0.0186 (6)	
C3C	1.0281 (3)	0.6376 (3)	0.54883 (14)	0.0176 (6)	
H3C	0.9909	0.7250	0.5238	0.021*	
C4C	0.9560 (3)	0.5771 (3)	0.61543 (15)	0.0189 (6)	
C5C	1.0120 (3)	0.4465 (3)	0.65207 (15)	0.0176 (6)	
C6C	1.1377 (3)	0.3812 (3)	0.62102 (15)	0.0186 (6)	
H6C	1.1755	0.2935	0.6454	0.022*	
C7C	0.7725 (3)	0.7672 (3)	0.61599 (16)	0.0259 (7)	
H7C1	0.8364	0.8367	0.6087	0.039*	
H7C2	0.6828	0.7955	0.6460	0.039*	
H7C3	0.7576	0.7605	0.5696	0.039*	
C8C	0.9994 (3)	0.2797 (3)	0.76102 (16)	0.0252 (7)	
H8C1	1.0156	0.1976	0.7396	0.038*	
H8C2	0.9389	0.2598	0.8087	0.038*	
H8C3	1.0891	0.3023	0.7650	0.038*	
Br1D	0.9822 (6)	0.9422 (4)	0.3907 (3)	0.0248 (3)	0.6246 (15)
Cl1D	0.667 (2)	1.065 (2)	0.4698 (8)	0.0265 (8)	0.6246 (15)
Br1H	0.6728 (14)	1.0541 (14)	0.4769 (5)	0.0265 (8)	0.3754 (15)
Cl1H	0.978 (2)	0.9528 (16)	0.3837 (11)	0.0248 (3)	0.3754 (15)
O1D	0.94378 (19)	1.42221 (19)	0.20683 (10)	0.0199 (4)	
O2D	0.69740 (19)	1.50728 (19)	0.27335 (10)	0.0196 (4)	
C1D	0.7583 (3)	1.1643 (3)	0.39183 (14)	0.0203 (7)	
C2D	0.8882 (3)	1.1176 (3)	0.35572 (15)	0.0190 (6)	
C3D	0.9541 (3)	1.2016 (3)	0.29312 (15)	0.0179 (6)	
H3D	1.0442	1.1693	0.2686	0.022*	
C4D	0.8889 (3)	1.3311 (3)	0.26678 (15)	0.0181 (6)	
C5D	0.7543 (3)	1.3787 (3)	0.30347 (15)	0.0170 (6)	
C6D	0.6913 (3)	1.2950 (3)	0.36545 (15)	0.0198 (6)	
H6D	0.6013	1.3266	0.3904	0.024*	
C7D	1.0845 (3)	1.3829 (3)	0.17138 (16)	0.0238 (7)	
H7D1	1.1459	1.3604	0.2057	0.036*	
H7D2	1.1163	1.4606	0.1327	0.036*	
H7D3	1.0873	1.3009	0.1515	0.036*	
C8D	0.5633 (3)	1.5601 (3)	0.31173 (16)	0.0234 (7)	
H8D1	0.4954	1.4951	0.3178	0.035*	
H8D2	0.5312	1.6517	0.2847	0.035*	
H8D3	0.5721	1.5693	0.3588	0.035*	

### Atomic displacement parameters (Å^2^)

**Table:**

	*U*^11^	*U*^22^	*U*^33^	*U*^12^	*U*^13^	*U*^23^
Br1A	0.0207 (6)	0.0204 (2)	0.0261 (8)	0.0010 (4)	0.0031 (4)	−0.0028 (4)
Cl1A	0.0260 (4)	0.016 (3)	0.0187 (17)	−0.0060 (16)	0.0003 (11)	0.0057 (14)
Br1E	0.0260 (4)	0.016 (3)	0.0187 (17)	−0.0060 (16)	0.0003 (11)	0.0057 (14)
Cl1E	0.0207 (6)	0.0204 (2)	0.0261 (8)	0.0010 (4)	0.0031 (4)	−0.0028 (4)
O1A	0.0190 (10)	0.0158 (10)	0.0253 (12)	0.0016 (8)	−0.0008 (9)	−0.0014 (9)
O2A	0.0220 (11)	0.0167 (11)	0.0232 (12)	−0.0037 (9)	0.0010 (9)	0.0032 (9)
C1A	0.0155 (14)	0.0203 (16)	0.0220 (16)	−0.0014 (12)	−0.0018 (12)	−0.0076 (13)
C2A	0.0204 (15)	0.0179 (15)	0.0162 (15)	−0.0072 (12)	−0.0028 (12)	−0.0037 (12)
C3A	0.0194 (15)	0.0141 (14)	0.0191 (16)	−0.0012 (12)	−0.0043 (12)	−0.0027 (12)
C4A	0.0162 (14)	0.0178 (15)	0.0212 (16)	−0.0025 (12)	−0.0044 (12)	−0.0070 (13)
C5A	0.0198 (15)	0.0190 (15)	0.0201 (16)	−0.0076 (12)	−0.0044 (13)	−0.0043 (13)
C6A	0.0217 (15)	0.0144 (15)	0.0222 (17)	−0.0029 (12)	−0.0048 (13)	−0.0036 (13)
C7A	0.0196 (15)	0.0186 (16)	0.0242 (17)	0.0010 (12)	−0.0055 (13)	−0.0024 (13)
C8A	0.0261 (17)	0.0180 (16)	0.0255 (18)	−0.0037 (13)	−0.0023 (14)	0.0027 (13)
Br1B	0.0320 (3)	0.0169 (9)	0.0225 (13)	0.0032 (7)	−0.0081 (7)	−0.0010 (7)
Cl1B	0.0267 (11)	0.0222 (16)	0.0212 (4)	−0.0091 (8)	−0.0005 (4)	0.0016 (7)
Br1F	0.0267 (11)	0.0222 (16)	0.0212 (4)	−0.0091 (8)	−0.0005 (4)	0.0016 (7)
Cl1F	0.0320 (3)	0.0169 (9)	0.0225 (13)	0.0032 (7)	−0.0081 (7)	−0.0010 (7)
O1B	0.0211 (11)	0.0248 (11)	0.0158 (11)	−0.0012 (9)	0.0018 (9)	0.0041 (9)
O2B	0.0227 (11)	0.0192 (11)	0.0184 (11)	0.0016 (9)	−0.0020 (9)	0.0006 (9)
C1B	0.0216 (15)	0.0193 (15)	0.0156 (15)	−0.0079 (12)	−0.0036 (12)	−0.0037 (12)
C2B	0.0220 (15)	0.0133 (14)	0.0224 (16)	−0.0013 (12)	−0.0099 (13)	−0.0027 (12)
C3B	0.0198 (15)	0.0223 (16)	0.0177 (16)	−0.0013 (12)	−0.0026 (13)	−0.0054 (13)
C4B	0.0186 (15)	0.0164 (15)	0.0150 (15)	−0.0044 (12)	−0.0026 (12)	−0.0018 (12)
C5B	0.0209 (15)	0.0160 (15)	0.0155 (15)	−0.0003 (12)	−0.0065 (12)	−0.0047 (12)
C6B	0.0174 (15)	0.0182 (15)	0.0173 (15)	−0.0037 (12)	−0.0033 (12)	−0.0034 (12)
C7B	0.0204 (16)	0.0349 (19)	0.0221 (18)	0.0021 (14)	0.0027 (13)	0.0019 (15)
C8B	0.0239 (16)	0.0218 (16)	0.0247 (17)	0.0039 (13)	−0.0021 (14)	−0.0058 (14)
Br1C	0.0183 (14)	0.0303 (3)	0.0202 (6)	−0.0011 (10)	0.0021 (7)	0.0009 (4)
Cl1C	0.0207 (9)	0.0226 (13)	0.0212 (9)	0.0018 (6)	−0.0004 (7)	−0.0075 (7)
Br1G	0.0207 (9)	0.0226 (13)	0.0212 (9)	0.0018 (6)	−0.0004 (7)	−0.0075 (7)
Cl1G	0.0183 (14)	0.0303 (3)	0.0202 (6)	−0.0011 (10)	0.0021 (7)	0.0009 (4)
O1C	0.0188 (10)	0.0202 (11)	0.0204 (11)	0.0029 (8)	0.0000 (9)	−0.0018 (9)
O2C	0.0240 (11)	0.0216 (11)	0.0179 (11)	−0.0002 (9)	0.0002 (9)	0.0016 (9)
C1C	0.0161 (14)	0.0202 (16)	0.0180 (16)	−0.0025 (12)	−0.0034 (12)	−0.0082 (13)
C2C	0.0198 (15)	0.0208 (16)	0.0154 (15)	−0.0059 (13)	−0.0029 (12)	−0.0026 (13)
C3C	0.0208 (15)	0.0150 (15)	0.0172 (16)	−0.0008 (12)	−0.0076 (13)	−0.0012 (12)
C4C	0.0171 (15)	0.0187 (15)	0.0212 (16)	−0.0035 (12)	−0.0021 (13)	−0.0056 (13)
C5C	0.0205 (15)	0.0165 (15)	0.0154 (15)	−0.0050 (12)	−0.0026 (12)	−0.0017 (12)
C6C	0.0228 (16)	0.0108 (14)	0.0222 (16)	−0.0010 (12)	−0.0073 (13)	−0.0017 (12)
C7C	0.0216 (16)	0.0233 (17)	0.0285 (18)	0.0037 (13)	−0.0049 (14)	−0.0025 (14)
C8C	0.0311 (17)	0.0217 (16)	0.0176 (16)	−0.0009 (13)	−0.0044 (14)	0.0034 (13)
Br1D	0.0298 (4)	0.0143 (6)	0.0241 (11)	0.0025 (5)	−0.0030 (6)	0.0014 (6)
Cl1D	0.0316 (9)	0.0224 (18)	0.0174 (17)	−0.0032 (11)	0.0017 (12)	0.0045 (10)
Br1H	0.0316 (9)	0.0224 (18)	0.0174 (17)	−0.0032 (11)	0.0017 (12)	0.0045 (10)
Cl1H	0.0298 (4)	0.0143 (6)	0.0241 (11)	0.0025 (5)	−0.0030 (6)	0.0014 (6)
O1D	0.0181 (10)	0.0182 (10)	0.0186 (11)	−0.0002 (8)	−0.0009 (9)	0.0012 (9)
O2D	0.0205 (11)	0.0156 (10)	0.0197 (11)	0.0017 (8)	−0.0031 (9)	−0.0021 (9)
C1D	0.0252 (16)	0.0177 (15)	0.0170 (16)	−0.0067 (13)	−0.0015 (13)	−0.0019 (13)
C2D	0.0231 (16)	0.0103 (14)	0.0249 (17)	−0.0015 (12)	−0.0085 (13)	−0.0032 (12)
C3D	0.0171 (14)	0.0161 (15)	0.0207 (16)	−0.0006 (12)	−0.0040 (12)	−0.0050 (12)
C4D	0.0215 (15)	0.0198 (16)	0.0137 (15)	−0.0072 (12)	−0.0033 (12)	−0.0018 (12)
C5D	0.0190 (15)	0.0140 (14)	0.0192 (16)	−0.0007 (11)	−0.0072 (12)	−0.0034 (12)
C6D	0.0204 (15)	0.0213 (16)	0.0177 (16)	−0.0030 (12)	−0.0021 (13)	−0.0059 (13)
C7D	0.0197 (16)	0.0231 (16)	0.0234 (17)	−0.0024 (13)	0.0028 (13)	−0.0029 (13)
C8D	0.0207 (16)	0.0224 (16)	0.0268 (18)	0.0050 (13)	−0.0066 (14)	−0.0085 (14)

### Geometric parameters (Å, °)

**Table:**

Br1A—C1A	1.890 (3)	Br1C—C2C	1.877 (5)
Cl1A—C2A	1.739 (8)	Cl1C—C1C	1.746 (10)
Br1E—C2A	1.862 (9)	Br1G—C1C	1.890 (5)
Cl1E—C1A	1.753 (11)	Cl1G—C2C	1.758 (10)
O1A—C4A	1.365 (3)	O1C—C4C	1.354 (3)
O1A—C7A	1.434 (3)	O1C—C7C	1.440 (3)
O2A—C5A	1.362 (3)	O2C—C5C	1.359 (3)
O2A—C8A	1.436 (3)	O2C—C8C	1.425 (3)
C1A—C2A	1.376 (4)	C1C—C2C	1.367 (4)
C1A—C6A	1.392 (4)	C1C—C6C	1.394 (4)
C2A—C3A	1.385 (4)	C2C—C3C	1.405 (4)
C3A—C4A	1.374 (4)	C3C—C4C	1.381 (4)
C3A—H3A	0.95	C3C—H3C	0.95
C4A—C5A	1.411 (4)	C4C—C5C	1.416 (4)
C5A—C6A	1.383 (4)	C5C—C6C	1.375 (4)
C6A—H6A	0.95	C6C—H6C	0.95
C7A—H7A1	0.98	C7C—H7C1	0.98
C7A—H7A2	0.98	C7C—H7C2	0.98
C7A—H7A3	0.98	C7C—H7C3	0.98
C8A—H8A1	0.98	C8C—H8C1	0.98
C8A—H8A2	0.98	C8C—H8C2	0.98
C8A—H8A3	0.98	C8C—H8C3	0.98
Br1B—C2B	1.879 (5)	Br1D—C2D	1.883 (4)
Cl1B—C1B	1.755 (10)	Cl1D—C1D	1.747 (10)
Br1F—C1B	1.867 (6)	Br1H—C1D	1.864 (7)
Cl1F—C2B	1.760 (11)	Cl1H—C2D	1.753 (11)
O1B—C4B	1.364 (3)	O1D—C4D	1.361 (3)
O1B—C7B	1.428 (3)	O1D—C7D	1.435 (3)
O2B—C5B	1.363 (3)	O2D—C5D	1.359 (3)
O2B—C8B	1.439 (3)	O2D—C8D	1.442 (3)
C1B—C2B	1.377 (4)	C1D—C2D	1.375 (4)
C1B—C6B	1.398 (4)	C1D—C6D	1.393 (4)
C2B—C3B	1.387 (4)	C2D—C3D	1.395 (4)
C3B—C4B	1.375 (4)	C3D—C4D	1.375 (4)
C3B—H3B	0.95	C3D—H3D	0.95
C4B—C5B	1.416 (4)	C4D—C5D	1.417 (4)
C5B—C6B	1.374 (4)	C5D—C6D	1.376 (4)
C6B—H6B	0.95	C6D—H6D	0.95
C7B—H7B1	0.98	C7D—H7D1	0.98
C7B—H7B2	0.98	C7D—H7D2	0.98
C7B—H7B3	0.98	C7D—H7D3	0.98
C8B—H8B1	0.98	C8D—H8D1	0.98
C8B—H8B2	0.98	C8D—H8D2	0.98
C8B—H8B3	0.98	C8D—H8D3	0.98
			
C4A—O1A—C7A	116.8 (2)	C4C—O1C—C7C	117.1 (2)
C5A—O2A—C8A	117.0 (2)	C5C—O2C—C8C	116.2 (2)
C2A—C1A—C6A	120.1 (2)	C2C—C1C—C6C	120.4 (2)
C2A—C1A—Cl1E	124.7 (8)	C2C—C1C—Cl1C	122.0 (8)
C6A—C1A—Cl1E	115.2 (8)	C6C—C1C—Cl1C	117.6 (8)
C2A—C1A—Br1A	121.1 (2)	C2C—C1C—Br1G	122.6 (4)
C6A—C1A—Br1A	118.8 (2)	C6C—C1C—Br1G	117.0 (3)
C1A—C2A—C3A	120.1 (2)	C1C—C2C—C3C	120.2 (2)
C1A—C2A—Cl1A	119.3 (6)	C1C—C2C—Cl1G	124.5 (6)
C3A—C2A—Cl1A	120.7 (6)	C3C—C2C—Cl1G	115.3 (6)
C1A—C2A—Br1E	123.3 (7)	C1C—C2C—Br1C	121.0 (3)
C3A—C2A—Br1E	116.6 (7)	C3C—C2C—Br1C	118.8 (3)
C4A—C3A—C2A	120.7 (3)	C4C—C3C—C2C	119.8 (3)
C4A—C3A—H3A	119.7	C4C—C3C—H3C	120.1
C2A—C3A—H3A	119.7	C2C—C3C—H3C	120.1
O1A—C4A—C3A	124.8 (3)	O1C—C4C—C3C	125.2 (3)
O1A—C4A—C5A	115.6 (2)	O1C—C4C—C5C	115.1 (2)
C3A—C4A—C5A	119.6 (3)	C3C—C4C—C5C	119.6 (3)
O2A—C5A—C6A	124.7 (3)	O2C—C5C—C6C	125.1 (3)
O2A—C5A—C4A	115.9 (2)	O2C—C5C—C4C	115.3 (2)
C6A—C5A—C4A	119.4 (3)	C6C—C5C—C4C	119.7 (3)
C5A—C6A—C1A	120.1 (3)	C5C—C6C—C1C	120.3 (3)
C5A—C6A—H6A	119.9	C5C—C6C—H6C	119.9
C1A—C6A—H6A	119.9	C1C—C6C—H6C	119.9
O1A—C7A—H7A1	109.5	O1C—C7C—H7C1	109.5
O1A—C7A—H7A2	109.5	O1C—C7C—H7C2	109.5
H7A1—C7A—H7A2	109.5	H7C1—C7C—H7C2	109.5
O1A—C7A—H7A3	109.5	O1C—C7C—H7C3	109.5
H7A1—C7A—H7A3	109.5	H7C1—C7C—H7C3	109.5
H7A2—C7A—H7A3	109.5	H7C2—C7C—H7C3	109.5
O2A—C8A—H8A1	109.5	O2C—C8C—H8C1	109.5
O2A—C8A—H8A2	109.5	O2C—C8C—H8C2	109.5
H8A1—C8A—H8A2	109.5	H8C1—C8C—H8C2	109.5
O2A—C8A—H8A3	109.5	O2C—C8C—H8C3	109.5
H8A1—C8A—H8A3	109.5	H8C1—C8C—H8C3	109.5
H8A2—C8A—H8A3	109.5	H8C2—C8C—H8C3	109.5
C4B—O1B—C7B	116.6 (2)	C4D—O1D—C7D	116.8 (2)
C5B—O2B—C8B	116.9 (2)	C5D—O2D—C8D	116.4 (2)
C2B—C1B—C6B	119.7 (2)	C2D—C1D—C6D	120.1 (2)
C2B—C1B—Cl1B	122.0 (8)	C2D—C1D—Cl1D	123.2 (7)
C6B—C1B—Cl1B	118.3 (8)	C6D—C1D—Cl1D	116.7 (7)
C2B—C1B—Br1F	120.8 (4)	C2D—C1D—Br1H	120.1 (5)
C6B—C1B—Br1F	119.5 (4)	C6D—C1D—Br1H	119.8 (5)
C1B—C2B—C3B	120.5 (3)	C1D—C2D—C3D	120.1 (3)
C1B—C2B—Cl1F	122.8 (8)	C1D—C2D—Cl1H	123.1 (8)
C3B—C2B—Cl1F	116.7 (8)	C3D—C2D—Cl1H	116.7 (8)
C1B—C2B—Br1B	120.2 (3)	C1D—C2D—Br1D	121.0 (3)
C3B—C2B—Br1B	119.4 (3)	C3D—C2D—Br1D	118.9 (3)
C4B—C3B—C2B	120.4 (3)	C4D—C3D—C2D	120.2 (3)
C4B—C3B—H3B	119.8	C4D—C3D—H3D	119.9
C2B—C3B—H3B	119.8	C2D—C3D—H3D	119.9
O1B—C4B—C3B	125.6 (3)	O1D—C4D—C3D	125.2 (3)
O1B—C4B—C5B	115.0 (2)	O1D—C4D—C5D	115.1 (2)
C3B—C4B—C5B	119.4 (3)	C3D—C4D—C5D	119.7 (3)
O2B—C5B—C6B	124.8 (3)	O2D—C5D—C6D	124.9 (3)
O2B—C5B—C4B	115.4 (2)	O2D—C5D—C4D	115.7 (2)
C6B—C5B—C4B	119.7 (3)	C6D—C5D—C4D	119.4 (3)
C5B—C6B—C1B	120.3 (3)	C5D—C6D—C1D	120.5 (3)
C5B—C6B—H6B	119.8	C5D—C6D—H6D	119.8
C1B—C6B—H6B	119.8	C1D—C6D—H6D	119.8
O1B—C7B—H7B1	109.5	O1D—C7D—H7D1	109.5
O1B—C7B—H7B2	109.5	O1D—C7D—H7D2	109.5
H7B1—C7B—H7B2	109.5	H7D1—C7D—H7D2	109.5
O1B—C7B—H7B3	109.5	O1D—C7D—H7D3	109.5
H7B1—C7B—H7B3	109.5	H7D1—C7D—H7D3	109.5
H7B2—C7B—H7B3	109.5	H7D2—C7D—H7D3	109.5
O2B—C8B—H8B1	109.5	O2D—C8D—H8D1	109.5
O2B—C8B—H8B2	109.5	O2D—C8D—H8D2	109.5
H8B1—C8B—H8B2	109.5	H8D1—C8D—H8D2	109.5
O2B—C8B—H8B3	109.5	O2D—C8D—H8D3	109.5
H8B1—C8B—H8B3	109.5	H8D1—C8D—H8D3	109.5
H8B2—C8B—H8B3	109.5	H8D2—C8D—H8D3	109.5
			
C6A—C1A—C2A—C3A	0.0 (4)	C6C—C1C—C2C—C3C	−0.3 (4)
Cl1E—C1A—C2A—C3A	−179.5 (12)	Cl1C—C1C—C2C—C3C	−177.9 (9)
Br1A—C1A—C2A—C3A	−179.2 (2)	Br1G—C1C—C2C—C3C	179.8 (4)
C6A—C1A—C2A—Cl1A	−179.4 (9)	C6C—C1C—C2C—Cl1G	−177.8 (8)
Cl1E—C1A—C2A—Cl1A	1.1 (15)	Cl1C—C1C—C2C—Cl1G	4.6 (12)
Br1A—C1A—C2A—Cl1A	1.4 (9)	Br1G—C1C—C2C—Cl1G	2.4 (9)
C6A—C1A—C2A—Br1E	−179.6 (10)	C6C—C1C—C2C—Br1C	178.6 (3)
Cl1E—C1A—C2A—Br1E	0.9 (16)	Cl1C—C1C—C2C—Br1C	1.0 (10)
Br1A—C1A—C2A—Br1E	1.2 (10)	Br1G—C1C—C2C—Br1C	−1.2 (5)
C1A—C2A—C3A—C4A	0.3 (4)	C1C—C2C—C3C—C4C	0.5 (4)
Cl1A—C2A—C3A—C4A	179.7 (9)	Cl1G—C2C—C3C—C4C	178.2 (7)
Br1E—C2A—C3A—C4A	180.0 (9)	Br1C—C2C—C3C—C4C	−178.5 (3)
C7A—O1A—C4A—C3A	−2.1 (4)	C7C—O1C—C4C—C3C	−0.5 (4)
C7A—O1A—C4A—C5A	178.3 (2)	C7C—O1C—C4C—C5C	179.0 (2)
C2A—C3A—C4A—O1A	179.9 (2)	C2C—C3C—C4C—O1C	179.2 (2)
C2A—C3A—C4A—C5A	−0.5 (4)	C2C—C3C—C4C—C5C	−0.3 (4)
C8A—O2A—C5A—C6A	−4.5 (4)	C8C—O2C—C5C—C6C	12.4 (4)
C8A—O2A—C5A—C4A	176.4 (2)	C8C—O2C—C5C—C4C	−167.7 (2)
O1A—C4A—C5A—O2A	−0.8 (4)	O1C—C4C—C5C—O2C	0.6 (4)
C3A—C4A—C5A—O2A	179.6 (2)	C3C—C4C—C5C—O2C	−179.8 (2)
O1A—C4A—C5A—C6A	−180.0 (2)	O1C—C4C—C5C—C6C	−179.5 (2)
C3A—C4A—C5A—C6A	0.4 (4)	C3C—C4C—C5C—C6C	0.1 (4)
O2A—C5A—C6A—C1A	−179.2 (2)	O2C—C5C—C6C—C1C	180.0 (2)
C4A—C5A—C6A—C1A	−0.1 (4)	C4C—C5C—C6C—C1C	0.1 (4)
C2A—C1A—C6A—C5A	−0.1 (4)	C2C—C1C—C6C—C5C	0.1 (4)
Cl1E—C1A—C6A—C5A	179.4 (11)	Cl1C—C1C—C6C—C5C	177.7 (9)
Br1A—C1A—C6A—C5A	179.1 (2)	Br1G—C1C—C6C—C5C	179.9 (4)
C6B—C1B—C2B—C3B	−0.2 (4)	C6D—C1D—C2D—C3D	0.9 (4)
Cl1B—C1B—C2B—C3B	177.6 (8)	Cl1D—C1D—C2D—C3D	179.0 (10)
Br1F—C1B—C2B—C3B	−178.3 (4)	Br1H—C1D—C2D—C3D	−178.2 (6)
C6B—C1B—C2B—Cl1F	177.8 (11)	C6D—C1D—C2D—Cl1H	−178.2 (10)
Cl1B—C1B—C2B—Cl1F	−4.4 (14)	Cl1D—C1D—C2D—Cl1H	−0.1 (14)
Br1F—C1B—C2B—Cl1F	−0.2 (12)	Br1H—C1D—C2D—Cl1H	2.7 (12)
C6B—C1B—C2B—Br1B	178.5 (4)	C6D—C1D—C2D—Br1D	179.3 (3)
Cl1B—C1B—C2B—Br1B	−3.7 (9)	Cl1D—C1D—C2D—Br1D	−2.6 (10)
Br1F—C1B—C2B—Br1B	0.4 (6)	Br1H—C1D—C2D—Br1D	0.2 (7)
C1B—C2B—C3B—C4B	−0.2 (4)	C1D—C2D—C3D—C4D	−0.6 (4)
Cl1F—C2B—C3B—C4B	−178.4 (10)	Cl1H—C2D—C3D—C4D	178.5 (10)
Br1B—C2B—C3B—C4B	−178.9 (4)	Br1D—C2D—C3D—C4D	−179.1 (3)
C7B—O1B—C4B—C3B	−0.6 (4)	C7D—O1D—C4D—C3D	−4.3 (4)
C7B—O1B—C4B—C5B	178.7 (2)	C7D—O1D—C4D—C5D	175.0 (2)
C2B—C3B—C4B—O1B	−179.6 (2)	C2D—C3D—C4D—O1D	179.2 (2)
C2B—C3B—C4B—C5B	1.2 (4)	C2D—C3D—C4D—C5D	−0.1 (4)
C8B—O2B—C5B—C6B	1.1 (4)	C8D—O2D—C5D—C6D	2.6 (4)
C8B—O2B—C5B—C4B	−178.6 (2)	C8D—O2D—C5D—C4D	−177.3 (2)
O1B—C4B—C5B—O2B	−1.3 (3)	O1D—C4D—C5D—O2D	1.1 (3)
C3B—C4B—C5B—O2B	178.0 (2)	C3D—C4D—C5D—O2D	−179.5 (2)
O1B—C4B—C5B—C6B	179.0 (2)	O1D—C4D—C5D—C6D	−178.8 (2)
C3B—C4B—C5B—C6B	−1.7 (4)	C3D—C4D—C5D—C6D	0.6 (4)
O2B—C5B—C6B—C1B	−178.4 (2)	O2D—C5D—C6D—C1D	179.7 (2)
C4B—C5B—C6B—C1B	1.2 (4)	C4D—C5D—C6D—C1D	−0.4 (4)
C2B—C1B—C6B—C5B	−0.3 (4)	C2D—C1D—C6D—C5D	−0.4 (4)
Cl1B—C1B—C6B—C5B	−178.2 (8)	Cl1D—C1D—C6D—C5D	−178.6 (9)
Br1F—C1B—C6B—C5B	177.8 (4)	Br1H—C1D—C6D—C5D	178.7 (6)
